# Carbon spheres@MnO_2_ core-shell nanocomposites with enhanced dielectric properties for electromagnetic shielding

**DOI:** 10.1038/s41598-017-16059-0

**Published:** 2017-11-20

**Authors:** Huiya Wang, Zhifan Zhang, Chengjun Dong, Gang Chen, Yude Wang, Hongtao Guan

**Affiliations:** School of Materials Science and Engineering, Yunnan University, 650091 Kunming, People’s Republic of China

## Abstract

Carbon spheres (CS)@MnO_2_ core-shell nanocomposites, with MnO_2_ nanoflakes uniformly coating at the surface of CS cores, were successfully synthesized by a facile water-bathing method. MnO_2_ amounts is estimated to be 24.7 wt% in CS@MnO_2_ nanocomposites. A high dielectric loss value and an electromagnetic shielding effectiveness of 16‒23 dB were observed for the CS@MnO_2_ in the frequency range of 8‒18 GHz, which is mainly attributed to the enhanced absorption loss. The incorporation of the CS with MnO_2_ improves the electrical conductivity. Meanwhile, the electromagnetic impendence matching has been significantly ameliorated. Moreover, the increasing interfaces between the CS and MnO_2_ facilitate the microwave attenuation as well. Thus, the electromagnetic shielding performances were greatly enhanced. Our findings provide an effective methodology for the synthesis of the CS@MnO_2_ core-shell nanocomposite for potential electromagnetic applications.

## Introduction

In recent years, the rapid development of electromagnetic wave communications and radar systems urgently expect the excellent electromagnetic interference (EMI) shielding or absorbing materials to fulfill the rising demands in environmental protection devices, anti-electromagnetic interference (EMI) coatings and self-concealing technologies^1^. Generally, two mechanisms for EMI shielding are widely acceptable, *e.g*., electromagnetic reflection and absorption. Regard to the electromagnetic wave (EMW) shields, the absorber plays a crucial role in improving the electromagnetic performances, which strongly depends on the crystallinity, morphology and granulometry of the absorber. Traditional absorbers, such as ferrites and conductive polymers, could make a strong absorption. However, the complicated morphological tailoring is needed. Besides, the thicknesses are often too thick for practical applications^2^. Comparatively, carbon materials have been extensively investigated in EMI shielding applications with the advantages of low density, excellent chemical resistance and diverse microstructures^3^. Yet, the high reflective characteristic of carbon materials caused by their larger conductivity and permittivity hampers their performances as effective EMW absorbers^4^.

More recently, core-shell structures have caught great interests as an effective route to improve the chemical homogeneity and hence enhance the functionality of the composite^5^. For instance, Dong *et al*. reported that polyaniline (PANi) coated Ni nanocomposites presented dual dielectric relaxation to improve the microwave absorption of Ni nanoparticulates. With a PANi content of 15.6 wt%, the nanocomposite exhibited an effective absorption bandwidth (reflection loss (*RL*) < −10 dB) in the range of 4.2‒18 GHz with a thickness of 2 to 6 mm^6^. The research from Singh *et al*. also indicated that the decoration of Co/Ni to single walled carbon nanotubes (SWCNTs) delivered an enhanced EMI shielding performance^7^. Similarly, Du *et al*. wrapped Fe_3_O_4_ microspheres with carbon shells, which displayed a wide absorption bandwidth of about 14 GHz (4.0‒18 GHz) with a variation in shell thickness from 1.5 to 5.0 mm^8^.

In this sense, it is highly speculated that coating carbon materials with a less conductive dielectric could be an effective strategy to improve the electromagnetic performances of carbon materials by modulating the impedance matching between carbon material and free space. Moreover, the core-shell structures introduce large specific surface areas and interfaces, which will arise more polarization effects to improve the EMI shielding properties^9^.

As a typical transition metal oxide, manganese dioxide (MnO_2_) has been intensively studied in the fields of catalyst, batteries and electrochemical capacitor electrodes, due to its unique advantages including natural abundance, cheap precursors, easy synthesis, and thermal stability^10–12^. Significantly, MnO_2_ has been proved to be a competitive candidate for EMW absorptions and shields^13–15^. Nevertheless, the weak electrical conductivity and poor magnetic loss requires morphological tailoring, ion doping or incorporating with conductive components to improve its electrochemical or electromagnetic properties^16,17^. Previously, our group synthesized α-MnO_2_ nanostructures using a facile water-bathing method. It was found that the MnO_2_/paraffin wax composites possessed great EMW absorption properties in the frequency range of 2‒18 GHz^14^, even in the low frequency range at low temperature^18^. Wang *et al*. hydrothermally synthesized MnO_2_ hollow microspheres consisted of MnO_2_ nanoribbons, showing enhanced EMW attenuation with an effective absorption bandwidth of about 4 GHz (14.0–18 GHz) in a thickness of 3 mm^19^. Ramaprabhu *et al*. reinforced polyvinylidene fluoride (PVDF) with both 5 wt.% MnO_2_ nanotubes (MNTs) and 1 wt.% functionalized multiwalled carbon nanotubes (f-MWCNT), which showed a EMI shielding effectiveness of ~20 dB in the whole X-band frequency range^20^.

In view of the above considerations, we design a core-shell nanostructure with relatively high conductivity carbon sphere (CS) core and MnO_2_ nanoflakes shell, which will provide better impedance matching as well as good EMW absorption based on MnO_2_, CS and their synergistic effects. The unique CS@MnO_2_ core-shell structure is expected to exhibit better electromagnetic performances. In brief, we successfully prepared CS@MnO_2_ core-shell nanostructure using a facile water-bathing method. The micro-structures are thoroughly characterized and then the EMI shielding performances are examined in the frequency range of 8‒18 GHz. Finally, possible explanations are discussed in great details.

## Results and Discussions

### Phase crystallinity

**Figure 1:**
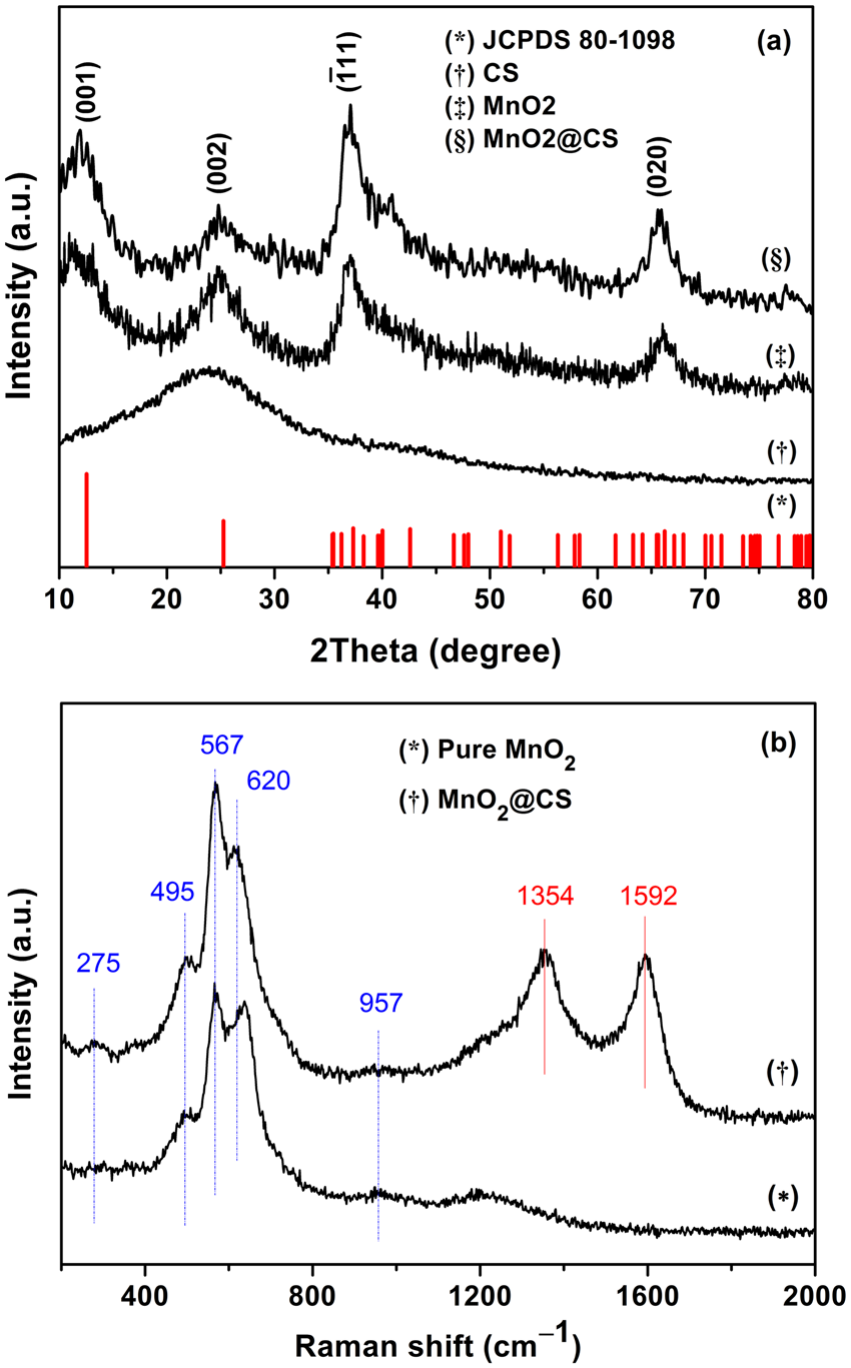
The XRD patterns (**a**) and Raman spectra (**b**) of the as-synthesized CS@MnO_2_ composite and pure MnO_2_ samples.

The crystal structure and phase purity of the as-prepared products were identified by XRD and Raman spectra. Figure 1a shows the typical XRD pattern of the samples including CS, MnO_2_ and CS@MnO_2_. All the detected diffraction peaks can be indexed to monoclinic potassium birnessite MnO_2_ (JCPDS 80–1098) regardless CS, which consists of 2D edge-shared [MnO_6_] octahedral layers with K^+^ cations and water molecules in the interlayer space^21^. Four diffraction peaks at 2*θ* of 12.6°, 25.2°, 37.3° and 65.6° correspond to the (001), (002), ($\overline{1}$ 11) and (020) basal reflections of MnO_2_, while the diffraction peaks at 11.6° and 24° are indexed to the diffraction of carbon materials^3^. Besides, no characteristic peaks of impurities are detected. The broadened peaks imply that the MnO_2_ is composed of small nanocrystals, which will be supported by the following TEM analysis.

To further compare the difference of MnO_2_ and CS@MnO_2_, the Raman spectra were obtained (Fig. 1b). The Raman peaks at 495, 567 and 636 cm^−1^ are derived from the birnessite-type MnO_2_, which belongs to A_g_ spectroscopic species originating from the breathing vibrations of [MnO_6_] octahedra within a birnessite-type framework. Generally, two Raman peaks at 567 cm^−1^ and 636 cm^−1^ are viewed as *ν*_3_(Mn-O) stretching vibration and the symmetric stretching vibration *ν*_2_(Mn-O) in [MnO_6_]^22^. Importantly, the Raman spectrum of CS@MnO_2_ appears similar features with pure MnO_2_, except a slight shift for the *ν*_2_(Mn-O) stretching frequency, which is ascribed to the interaction between the MnO_2_ and carbon materials^23^. Characteristic D band and G band peaks at 1354 cm^−1^ and 1592 cm^−1^, confirm the existence of the CS in the composites, which agrees well with previous reports^11,21^.

**Figure 2:**
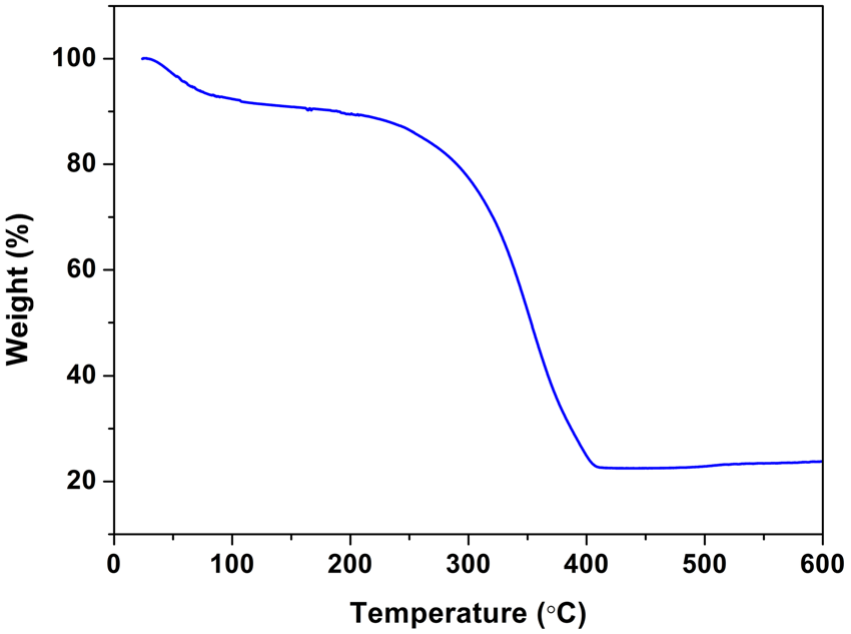
The TGA curve of the CS@MnO_2_ composite.

To estimate the MnO_2_ amounts in CS@MnO_2_ nanocomposite, a TGA analysis has been performed in air. Totally, the weight loss of 76.3 wt% is clearly observed from room temperature to 400 °C in Fig. 2, indicating the complete consumption of CS at about 400 °C^21,24^. Therefore, the MnO_2_ amounts is estimated to be 24.7 wt% in the CS@MnO_2_ composites.

### Microstructures

**Figure 3:**
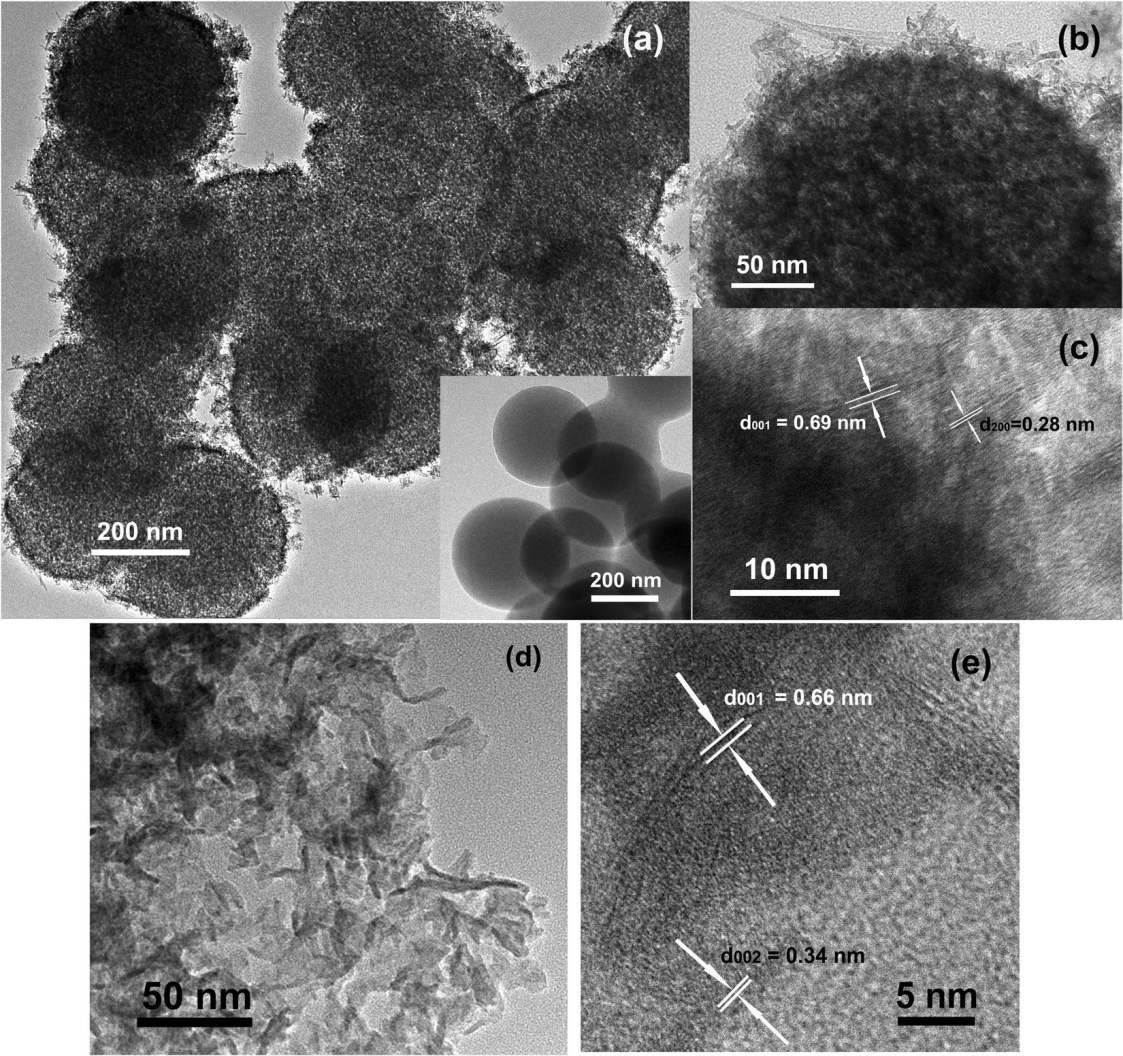
The TEM (**a**,**b**,**d**) and HETEM (**c**,**e**) of the CS@MnO_2_ composite (**a**,**b**,**c**) and pure MnO_2_ (**d**,**e**) samples.

The TEM and HRETM images of the CS@MnO_2_ and MnO_2_ nanostructures are displayed in Fig. 3. From Fig. 3a and b, it can be seen that the CS core (about 300 nm in diameter in the inset of Fig. 3a) is uniformly covered with a layer of about several nanometers in thickness. The MnO_2_ coating is in the form of tiny nanoflakes. As is known, carbon spheres synthesized with hydrothermally treated glucose usually have large functional groups on their surfaces, which produces plentiful active sites to favor MnO_2_ coating^25,26^. Figure 3c illustrates the MnO_2_ shell in CS@MnO_2_ by HRTEM, which reveals the interplanar spacings of 0.69 nm and 0.28 nm, matching well with the (001) and (200) crystal planes of birnessite MnO_2_. In the case of pure MnO_2_, the MnO_2_ is in the form of nanoflakes with dimensions of 30–50 nm (Fig. 3d). The HRTEM shows the interplanar spacings of 0.66 nm and 0.34 nm, as depicted in Fig. 3e, corresponding to the (001) and (200) planes of birnessite MnO_2_.

In short, these analyses confirm the formation of CS@MnO_2_ core-shell structure by a facile water-bathing method. The unique structure of CS@MnO_2_ makes it a favorable candidate for electromagnetic shielding.

### Electromagnetic results

**Figure 4:**
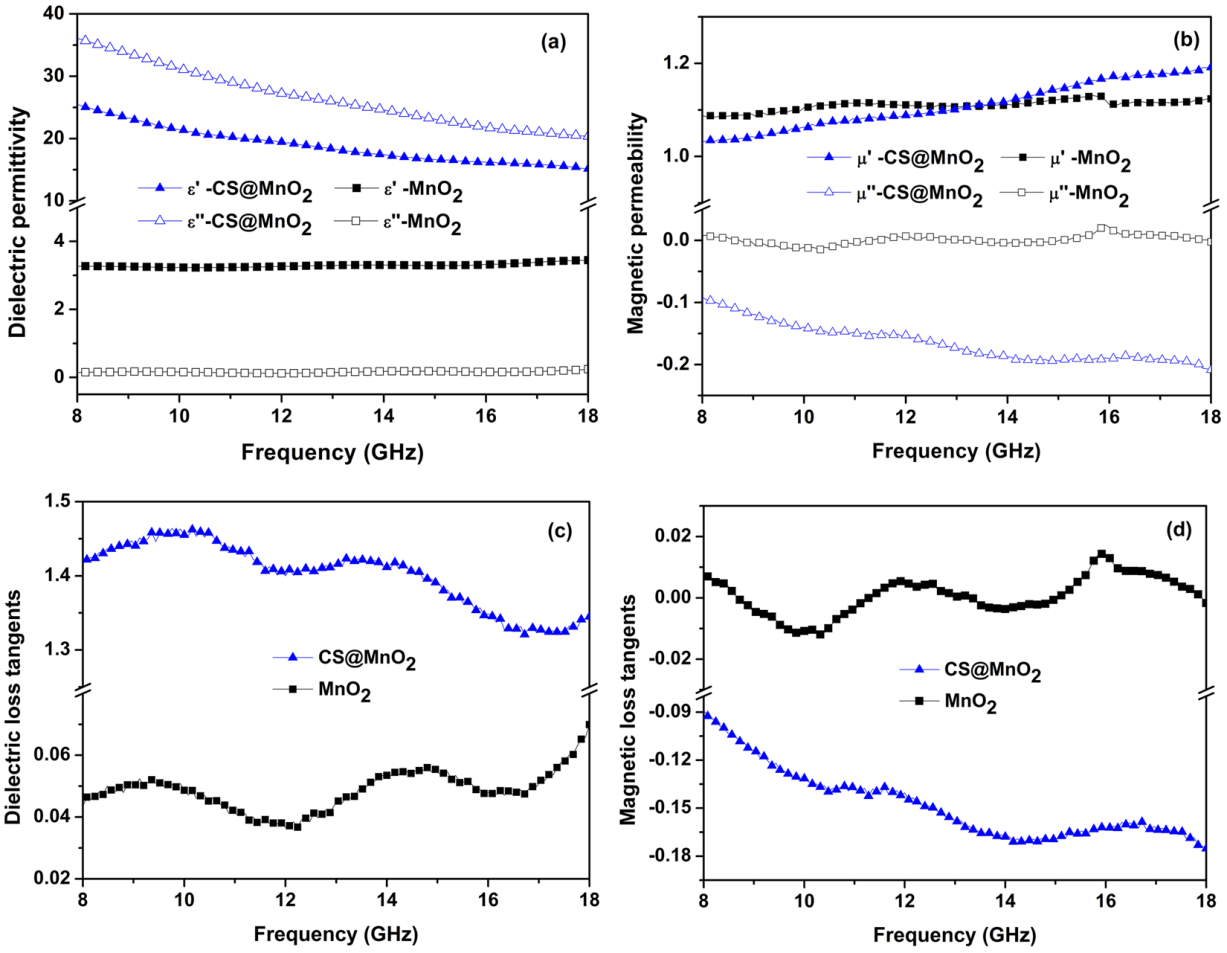
The frequency-dependent dielectric permittivity (**a**), magnetic permeability (**b**) and loss tangents (**c**,**d**) of the CS@MnO_2_ composite and pure MnO_2_ samples.

As we all know, the electromagnetic shielding performances are closely associated with their dielectric (${\varepsilon }_{{\rm{r}}}={\varepsilon ^{\prime} }_{{\rm{r}}}-{\rm{j}}{\varepsilon ^{\prime\prime} }_{{\rm{r}}}$) and magnetic (${\mu }_{{\rm{r}}}={\mu ^{\prime} }_{{\rm{r}}}-{\rm{j}}{\mu ^{\prime\prime} }_{{\rm{r}}}$) properties, as plotted in Fig. 4. It is found a decreasing *ε*_r_ with the increasing frequency from 8 to 18 GHz for the CS@MnO_2_ (Fig. 4a). On the contrary, an increasing *μ*_r_ is observed for the same sample (Fig. 4b). Nevertheless, both the *ε*_r_ and *μ*_r_ values of pure MnO_2_ are almost maintained. For instance, the real part of *ε*_r_ decreases from 25.5 at 8 GHz to 15.2 at 18 GHz, while the imaginary part drops from 36.1 at 8 GHz to 20.4 at 18 GHz, which may be favorable to the electromagnetic shielding performances. Particularly, great difference is observed in imaginary parts of *μ*_r_ between MnO_2_ and CS@MnO_2_, as shown in Fig. 4b. Compared to the almost steady imaginary *μ*_r_ values (1.05–1.20) of MnO_2_, the CS@MnO_2_ shows negative imaginary *μ*_r_ from −0.09 to −0.21. This phenomenon is not common in ordinary materials; however, it has been intensively reported for certain designed nanostructures such as hollow cobalt nanochains^27^, Fe/Al_2_O_3_^28^, and Ni/Al_2_O_3_^29^.

To investigate the electromagnetic loss characteristics of the CS@MnO_2_ core-shell composites, the dielectric and magnetic dissipation factors tan*δ*_*e*_ = *ε*″/*ε*′ and tan *δ*_*m*_ = *μ*″/*μ*′ are calculated, as expressed in Fig. 4. The values of tan*δ*_*e*_ for CS@MnO_2_ (>1.3) are much larger than pure MnO_2_ (0.03‒0.06), which comes from the synergic results of electronic polarization, interfacial polarization, dipole relaxation and natural resonance. Besides, the unique core-shell structure is also beneficial to the great tan*δ*_*e*_^7^. Under an external electromagnetic field, the charges accumulate at the interfaces between the MnO_2_ tiny dendrites and the carbon spheres to form enormous diploes, hence it could contribute to the dielectric loss by causing pronounced interfacial polarization. Owing to the surface anisotropy field, the materials in nano-scale may produce higher anisotropy energy to favor dielectric loss^30^. Moreover, great performances in electrochemical and electromagnetic fields^31–33^ were reported for MnO_2_ nanostructures with sheet- or flake-like shapes because of larger specific surface areas and better surficial activities.

As to tan*δ*_*m*_, pure MnO_2_ shows a tan*δ*_*m*_ less than 0.02, while the CS@MnO_2_ composite gives negative values due to its negative imaginary *μ*_r_, as demonstrated in Fig. 4d. Similar to tan*δ*_*e*_, two obvious peaks at around 10 GHz and 14 GHz for both MnO_2_ and CS@MnO_2_ are also observed, which is related to the resonances in *ε*_r_^1,34^. Based on the above results, CS@MnO_2_ core-shell is believed to exhibit excellent electromagnetic shielding performances over pure MnO_2_ in terms of higher dielectric and magnetic loss properties.

### EMI shielding effectiveness

The shielding effectiveness (*SE*) towards EMI was calculated by the relation:

1 $$SE=20\,{\rm{lg}}({E}_{{\rm{in}}}/{E}_{{\rm{tr}}})$$

According to the Schelkunoff’s Equation, the *SE* based on transmission line theory is expressed as^1,7^:

2 $$SE=S{E}_{{\rm{A}}}+S{E}_{{\rm{R}}}+S{E}_{{\rm{M}}}({\rm{dB}})$$

Where, *SE*_A_, *SE*_R_ and *SE*_M_ represent shielding contributions from absorption, reflection and multiple reflection, respectively. Generally, the *SE*_M_ can be ignored for all practical purposes when the *SE* is larger than 10 dB, and *SE*_R_ and *SE*_A_ are thus given using the following equations^2^:

3 $$S{E}_{{\rm{R}}}=-10\,{\rm{lg}}(1-R)$$

4 $$S{E}_{{\rm{A}}}=-10\,{\rm{lg}}(T/(1-R))$$

Where, *T* and *R* are the transmission coefficient and reflection coefficient, which is obtained by the following equations^35,36^,

5 $$T={|\frac{{E}_{{\rm{tr}}}}{{E}_{{\rm{in}}}}|}^{2}={|{S}_{21}|}^{2}={|{S}_{12}|}^{2}$$

6 $$R={|\frac{{E}_{{\rm{re}}}}{{E}_{{\rm{in}}}}|}^{2}={|{S}_{11}|}^{2}={|{S}_{22}|}^{2}$$

Where the *E*_in_, *E*_re_ and *E*_tr_ are the electric intensity of the incident, reflected and transmission waves, respectively.

**Figure 5:**
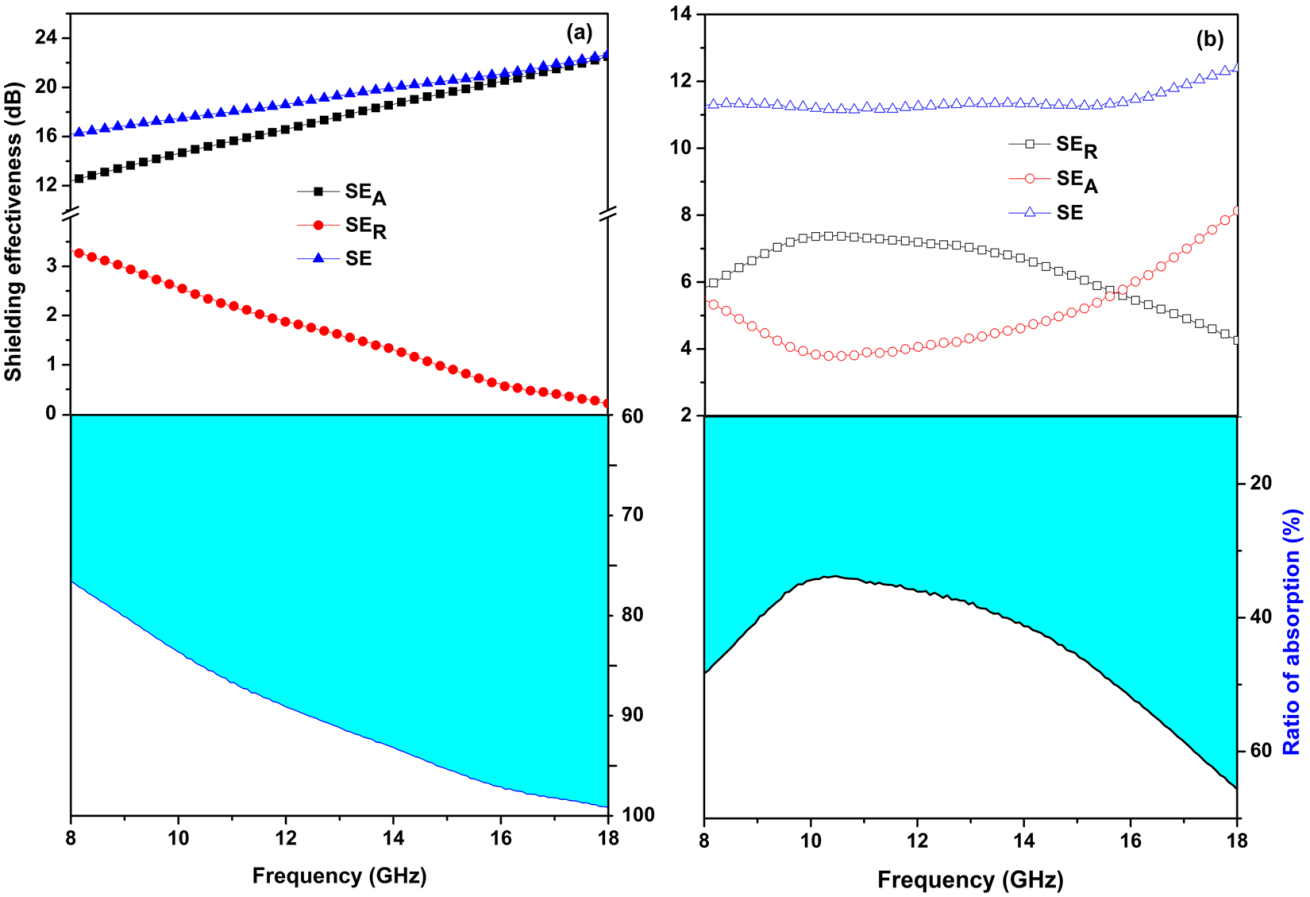
The electromagnetic shielding effectiveness of the CS@MnO_2_ composite (**a**) and pure MnO_2_ (**b**) samples.

The obtained *SE* of MnO_2_ nanoflakes and CS@MnO_2_ core-shell nanostructure with a thickness of 2 mm are shown in Fig. 5. Compared to the small *SE* values of MnO_2_ (11.3‒12.3 dB) (Fig. 5b), CS@MnO_2_ displays an increasing *SE* from 16.2 dB to 22.8 dB as the frequency increasing from 8 GHz to 18 GHz, indicating an improvement of the shielding proficiency of the incident EMW from 85% to 93% (Fig. 5a). The *SE* mainly arises from *SE*_A_, especially at high frequency bands. For example, the *SE*_A_ values at 8 GHz and 18 GHz are 12.4 and 22.5 dB, which accounts for 76.5% and 98.7% of the total *SE*. The excellent EMI shielding properties of CS@MnO_2_ is plausibly explained by the unique core-shell structure and the synergistic effect. Because of the poor conductivity of MnO_2_, a better impedance matching with the air is achieved. In the case of carbon materials, great EMW reflection on the surface may occur due to the impedance mismatching caused by the relatively higher electrical conductivity. When the incident EMW transmits into the CS@MnO_2_ core-shell nanostructure, the better impedance matching between the air and MnO_2_ will guide the wave into the interior of the structure to reach the carbon surface. The EMW is partially attenuated by the electric loss from carbon core. On the other hand, the reflected wave from the CS surface is absorbed again by the MnO_2_ shell. Hence, the effective complementarities between the dielectric loss and the magnetic loss, originating from the synergistic effect of the CS cores and the MnO_2_ nanoflaky shells, also endow CS@MnO_2_ with a better absorption^37^. As listed in Table 1, the SE values are clearly comparable to those reported MnO_2_-based EMI shielding materials.

**Table 1:** Comparison of the electromagnetic SE values of some MnO_2_ composites.

Materials/Composites	MnO_2_ loading	Frequency (GHz)	Thickness	SE (dB)	ref.
MnO_2_ nanotubes/PVDF	5 wt.%	8‒12	1 mm	9‒13	20
MnO_2_ nanotubes/MWCNT/PVDF	2 wt.%	8‒12	1 mm	18‒22	20
Nano β-MnO_2_/wax	30 wt.%	8.2‒12.4	2 mm	21.6‒22.5	44
MnO_2_/cement composite	10 wt.%	8‒13	10 mm	4‒9	45
MnO_2_/graphene nanoribbons composite	53 wt.%	8	2 mm	25	46
MnO_2_/porous carbon foam composite	2 wt.%	8.2‒12.4	2.5 mm	30‒33.6	47
	4 wt.%	8.2‒12.4	2.5 mm	36‒39	
MnO_2_/PANI films	25 wt.% to aniline	8.2‒12.4	81 ± 3 μm	24‒28	48
		12.4‒18	81 ± 3 μm	25‒27	
CS@MnO_2_ core-shell structure	~25 wt.%	8‒18	2 mm	16‒23	This work

**Figure 6:**
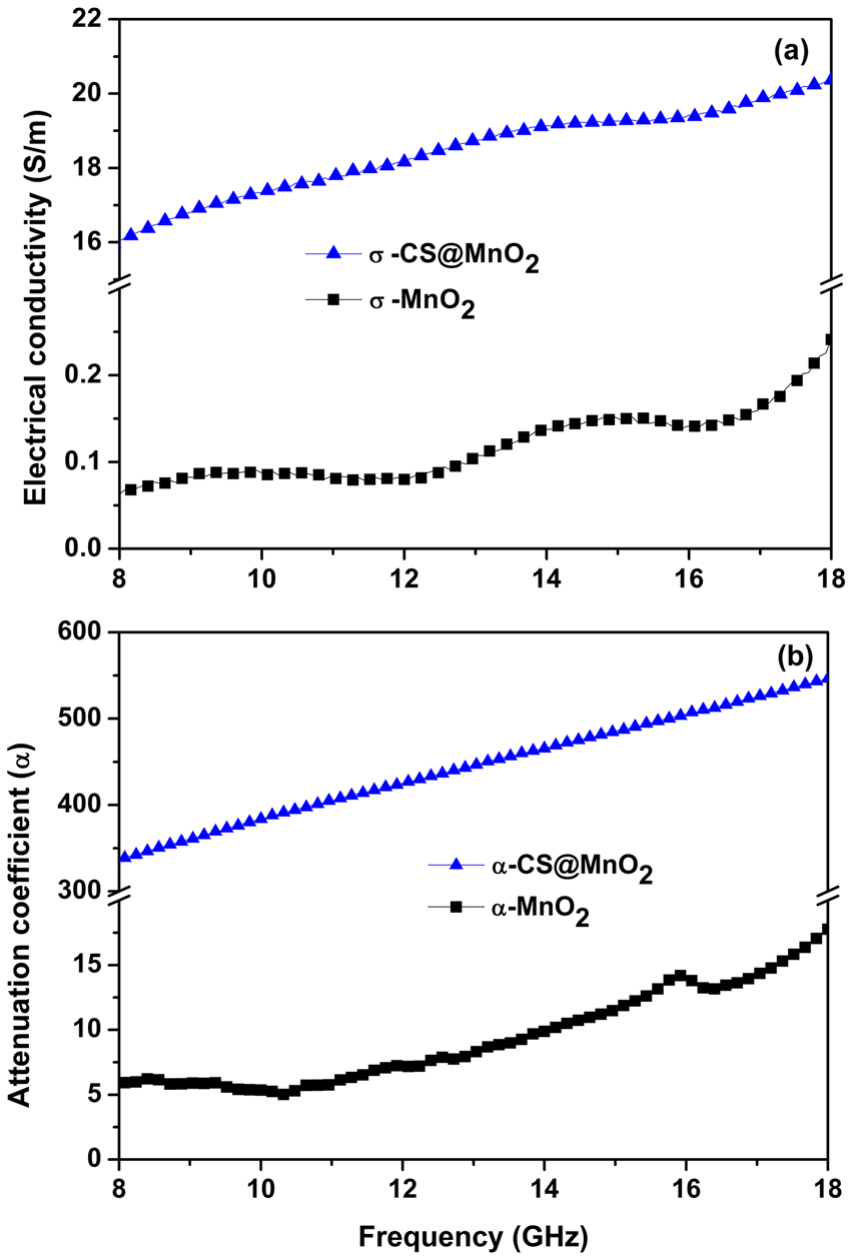
The frequency-dependent electrical conductivity (**a**) and attenuation coefficients (**b**) of the CS@MnO_2_ composite and pure MnO_2_ samples.

To completely explore the underlying electromagnetic mechanisms of the CS@MnO_2_ core-shell nanostructure, the electrical conductivity (σ), the dielectric polarization as well as magnetization are totally examined. Based on the measured electromagnetic parameters, the frequency dependent electrical conductivity of the pure MnO_2_ and CS@MnO_2_ are calculated from Equation (7), as displayed in Fig. 6a.

7 $$\sigma =2\pi \,f{\varepsilon ^{\prime\prime} }_{{\rm{r}}}\,{\varepsilon }_{0}$$

Conductivity of 0.06–0.24 S/m is obtained for pure MnO_2_ from 8 GHz to 18 GHz. However, bigger electrical conductivity (about 16–20.11 S/m) of CS@MnO_2_ is indicated in Fig. 6a at the same frequency range.

The electromagnetic attenuation coefficient (α) is calculated based on *ε*_r_ and *μ*_r_ values by the Equation (8).

8 $$\alpha =\frac{\pi \,f}{c}\sqrt{{\mu ^{\prime} }_{r}{\varepsilon ^{\prime} }_{r}}\sqrt{\tan \,{\delta }_{e}\,\tan \,{\delta }_{m}-1+\sqrt{1+{\tan }^{2}{\delta }_{e}+{\tan }^{2}{\delta }_{m}+{\tan }^{2}{\delta }_{e}{\tan }^{2}{\delta }_{m}}}$$

here, *f* and *c* are the frequency and electromagnetic wave velocity in free space, respectively. Although both samples show increasing α values with frequency, the CS@MnO_2_ core-shell structure clearly show much higher values, as shown in Fig. 6b, suggesting a suitable explanation of excellent *SE*.

In order to explore the intrinsic dielectric and magnetic response, the Debye dielectric relaxation model is adopted to analyze the mechanisms of the dielectric loss. According to the Debye dipolar relaxation, the dielectric permittivity in complex form can be expressed as^38^:

9 $$\varepsilon ^{\prime} ={\varepsilon }_{\infty }+\frac{{\varepsilon }_{s}-{\varepsilon }_{\infty }}{1+{(2\pi f)}^{2}{\tau }^{2}},\varepsilon ^{\prime\prime} =\frac{2\pi f\tau \,\,({\varepsilon }_{s}-{\varepsilon }_{\infty })}{1+{(2\pi f)}^{2}{\tau }^{2}}$$

where *f* is the frequency, *τ* is the relaxation time, *ε*_s_ and *ε*_∞_ are the dielectric constant in static and optical frequency, respectively. Accordingly, the relationship between ε′ and ε″ is further expressed as following^39^:

10 $${(\varepsilon ^{\prime} -\frac{{\varepsilon }_{s}+{\varepsilon }_{\infty }}{2})}^{2}+{(\varepsilon ^{\prime\prime} )}^{2}={(\frac{{\varepsilon }_{s}-{\varepsilon }_{\infty }}{2})}^{2}$$

**Figure 7:**
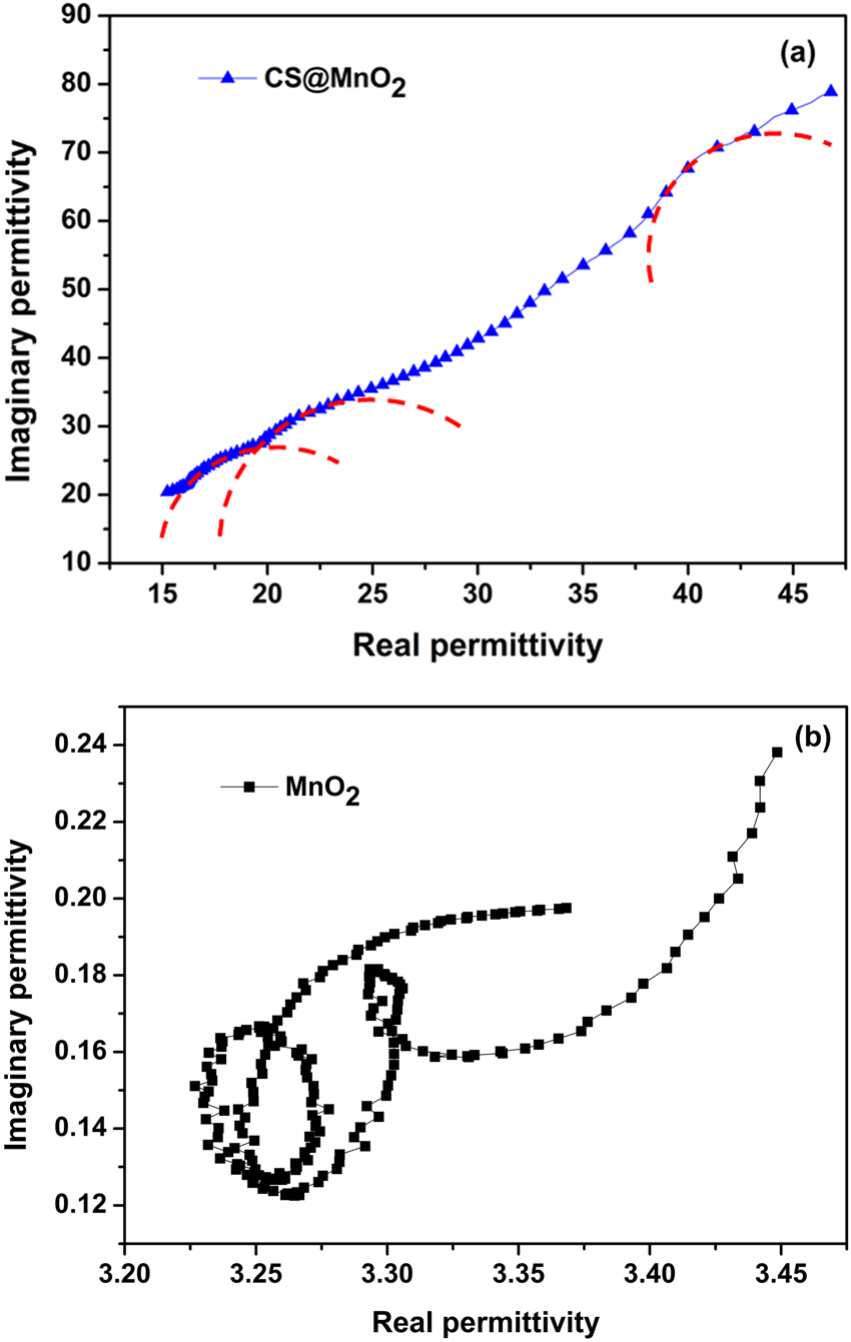
The Cole–Cole semicircles for the CS@MnO_2_ composite (**a**) and pure MnO_2_ (**b**) (the red lines indicate the fitting curve).

Therefore, the curve of *ε*′ versus *ε*″ is a single semicircle, denoting as Cole–Cole semicircle. Each semicircle generally corresponds to one type of Debye relaxation process. From Fig. 7a, three semicircles are detected for the CS@MnO_2_, expressing several dielectric loss mechanisms such as interfacial polarization, space charge polarization besides dielectric relaxation. Moreover, the conductance loss caused by the improved electrical conductivity may also contribute greatly^38^. The Cole–Cole semicircle curve of pure MnO_2_, as is in Fig. 7b, exhibits distorted and complicated shape, implying the existence of diverse dielectric loss processes^40^.

**Figure 8:**
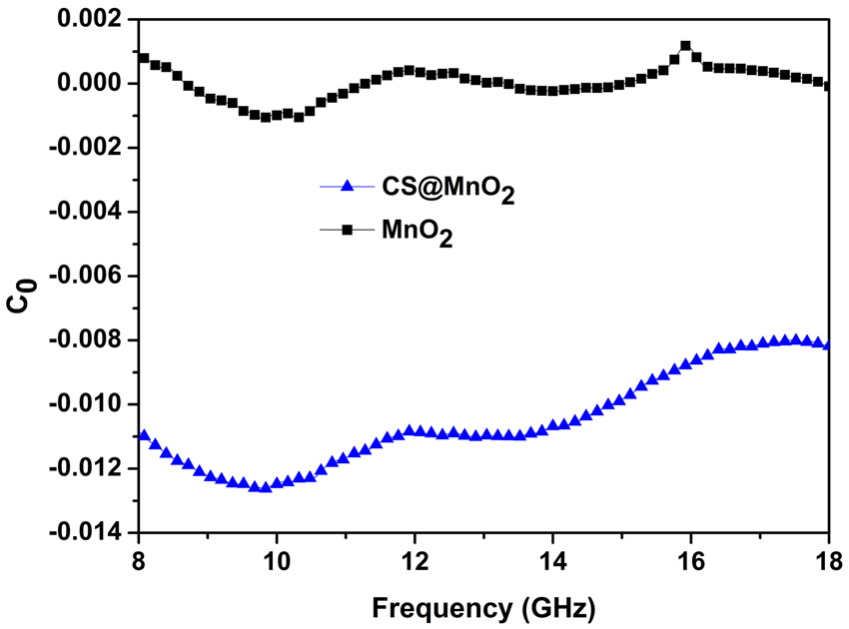
The eddy current loss domination curves of the CS@MnO_2_ composite (**a**) and pure MnO_2_ (**b**) samples.

As discussed above, magnetic loss also plays significant role in the electromagnetic attenuation process. In particular, the contributions of eddy current loss, as approximately described by Equation (11)^41^, to magnetic loss is studied.

11 $${\mu ^{\prime\prime} }_{{\rm{r}}}=\frac{2\pi \,f}{3}\sigma {\mu }_{0}{({\mu ^{\prime} }_{r})}^{2}{d}^{2}$$

where, *d* and *μ*_0_ are the particle diameter and the magnetic permeability of free space. For a magnetic material, the parameter *C*_0_ = *μ*″(*μ*′)^−2^(*f*)^−1^ is often used to determine its eddy current loss domination. As shown in Fig. 8, the *C*_0_ fluctuates markedly in the range of 8‒18 GHz, indicating complicated loss mechanisms other than only eddy current loss, such as magnetic hysteresis loss, domain-wall displacement loss and natural resonance^41^. The better magnetic loss of the CS@MnO_2_ could be ascribed to its unique core-shell structure. Liu *et al*. reported that the high-frequency performances of magnetic composites were enhanced by the separation of conductive particles with an insulating shell^42^. The incorporation of CS with MnO_2_ nanoflaky particles improves the electrical conductivity greatly, and thus results in larger eddy current densities, which produce significant eddy losses in an alternating electromagnetic field. Moreover, the time lag of the magnetization vector causes magnetic loss as well. As the frequency increases, the motion of the magnetization cannot keep up with the alternation of the applied electromagnetic field, leading to the increase of the imaginary *μ*_r_, and thus the magnetic loss^42^, as shown in Fig. 4.

Taken together, we could imagine the possible electromagnetic shielding mechanism for the CS@MnO_2_ core-shell based on the native natures of CS and MnO_2_ and the synergistic effect. When an incident EMW arrives at the surface of the CS@MnO_2_ composites, most incident wave will transmit through the thin MnO_2_ shell due to the improved impendence matching. At the interface between CS and MnO_2_, partial wave is attenuated by CS in the form of dielectric loss and magnetic eddy loss. The rest is reflected back to the shell again and attenuated by the MnO_2_ nanoflakes. Moreover, CS provides places for tiny MnO_2_ nanoflakes coating, enormous interfaces exists between the CS core and MnO_2_ shell, producing large amounts of polarization sites. These active sites, along with the dangling bonded atoms as well as unsaturated coordinations, derive remarkable interface polarization and obvious attenuation^43^. Last but not least, the eddy currents from natural resonance contribute significantly towards better absorption performances^7^ due to enhanced surface anisotropy of the tiny MnO_2_. Our results suggest that the CS@MnO_2_ core-shell composite can be taken as a potential EMI shielding material with absorption as the dominant shielding efficiency.

## Conclusions

In summary, CS@MnO_2_ core-shell structured composite with MnO_2_ (24.7 wt%) nanoflakes coated on the surface of the carbon sphere cores has been developed with a facile water-bathing method. The CS@MnO_2_ composite shows enhanced dielectric loss and EMI shielding performances in the frequency range of 8‒18 GHz. A shielding effectiveness of 16‒23 dB is obtained with a thickness of 2 mm. It is found that the shielding efficiency is dominantly from absorption. The incorporation of MnO_2_ nanoflaky shells with spherical carbon cores ameliorates the electrical conductivity and the impendence matching of the CS@MnO_2_ composites. Thus, the electromagnetic shielding properties are greatly improved.

## Methods

### Synthesis of CS@MnO_2_ core-shell composites

All chemicals were purchased from commercial sources with chemical grade and used without any further purification.

CS was prepared according to a typical hydrothermal method. Typically, 50 mL glucose solution with the concentration of 0.5 mol/L was transferred into a Teflon-lined autoclave and the hydrothermal reaction was conducted at 180 °C for 10 h. After cooled down to room temperature naturally, the brown product was collected by centrifugation and washed several times with water and absolute ethanol, and then dried at 100 °C for 10 h.

To produce the MnO_2_ coating, 0.1 g as-prepared CS was dispersed in 100 mL deionized water with the aid of ultrasonication for 2 h. Then 0.6 g KMnO_4_ was dissolved into the previous solution with vigorous magnetic stirring for 30 min. Thereafter, the mixture solution was reacted at 80 °C for 6 h in a water-bathing. The obtained suspension was centrifuged and repeatedly washed with water and absolute ethanol, then dried at 60 °C. For comparison, another sample was obtained following the same method without CS.

### Crystal and morphology characterizations

The crystal structures were investigated by powder X-Ray diffraction (XRD) on Rigaku TTR-III diffractometer with the Cu kα radiation (*λ* = 0.15418 nm) in the diffraction range of 2*θ* = 10–80°. The Raman spectra were recorded on a Reinshaw inVia Raman microscope with a power output of 3 mW under the laser excitation of 514 nm. The thermal decomposition analysis was determined on an American TA Q500 TGA thermal analyzer in air with a heating rate of 10 °C/min. The morphologies of the samples were observed by transmission electron microscopy (TEM, JEM-2100) with an accelerating voltage of 200 kV. To make a uniform dispersion of the products, the samples for TEM analysis were pretreated by dispersing it in absolute alcohol with a few seconds sonication and then dropped on a copper grid supported with amorphous carbon film.

### Electromagnetic characterization

The dielectric permittivity and magnetic permeability, as well as the electromagnetic S parameters (*S*_11_, *S*_12_, *S*_21_ and *S*_22_) were measured at room temperature from 8 to 18 GHz by a vector network analyzer (VNA, Agilent Technologies, N5230A). The product was uniformly mixed with paraffin wax in mass ratios of 50 wt% and pressed into a 2-mm-thick ring sample with an inner diameter of 3.04 mm and outer diameter of 7.00 mm.
